# Elevated Platelet to Lymphocyte Ratio Is Associated with Poor Survival Outcomes in Patients with Colorectal Cancer

**DOI:** 10.1371/journal.pone.0163523

**Published:** 2016-09-22

**Authors:** Xiaobin Gu, Xian-Shu Gao, Shangbin Qin, Xiaoying Li, Xin Qi, Mingwei Ma, Hao Yu, Shaoqian Sun, Dong Zhou, Wen Wang, Wei Xiong

**Affiliations:** 1 Department of Radiation Oncology, Peking University First Hospital, Peking University, Beijing, China; 2 Tangshan People’s Hospital, Hebei, China; The Ohio State University, UNITED STATES

## Abstract

Platelet to lymphocyte ratio (PLR) is a parameter reflecting inflammatory responses in patients with cancer. Several studies have investigated the prognostic value of PLR in patients with colorectal cancer (CRC); however, the results are controversial. Thus, we carried out a meta-analysis to evaluate the association between PLR and CRC prognostication. Relevant articles were retrieved through PubMed, Embase, and Web of Science, and pooled hazard ratio (HR) and 95% confidence interval (CI) were computed by using STATA V.12.0. Both the random-effects model and fixed-effects model were utilized. A total of 13 studies (14 cohorts) with 8,601 patients were included in the meta-analysis. Pooled HRs and 95% CIs demonstrated that increased PLR predicted poor overall survival (OS) (HR = 1.81, 95%CI:1.42–2.31, p<0.001; I^2^ = 65%, P_h_ = 0.002), disease-free survival (DFS) (HR = 1.84, 95%CI:1.22–2.76, p = 0.003; I^2^ = 78.3%, P_h_<0.001) and recurrence-free survival (RFS) (HR = 1.84, 95%CI:1.41–2.41, p<0.001; I^2^ = 0, P_h_ = 0.686), although this was not the case for cancer-specific survival (CSS) (HR = 1.75, 95%CI:0.59–5.17, p = 0.309; I^2^ = 66.2%, P_h_ = 0.085) or time to recurrence (TTR) (HR = 1.21 95%CI:0.62–2.36, p = 0.573;I^2^ = 58.4%, P_h_ = 0.121). Subgroup analysis showed that PLR enhanced the prognostic value for OS in Caucasian patients, in small sample studies and for metastatic disease; however, this was not the case with rectal cancer. Furthermore, elevated PLR predicted reduced DFS in Caucasians and not in Asians. In conclusion, our meta-analysis showed that high PLR was a significant biomarker for poor OS, DFS, and RFS in patients with CRC; however, it had no association with CSS or TTR.

## Introduction

Colorectal cancer (CRC) is ranked as the third most commonly diagnosed cancer type and the fourth most frequent cause of cancer-related deaths around the world[1]. In the United States, CRC accounts for 8% of new cancer cases and 8% of cancer deaths in men and women[2]. Significant progress has been achieved in the past two decades to improve the clinical outcomes of CRC, including the approval of several therapeutic agents for chemotherapy and targeted therapy [3]. Despite this, 24%-41% of patients die within 5 years following a surgical resection with curative intent, and 56%-78% of patients die within 2 years after palliative resection [4]. Therefore, it is necessary to identify novel and readily available prognostic biomarkers for risk stratification and to predict treatment efficiency in CRC.

Inflammation has been indicated to serve a pivotal role in cancer development[5]. Inflammatory responses can facilitate tumor progression in different stages, including initiation, proliferation, angiogenesis, invasion, and metastasis [6, 7]. In recent years, blood based inflammatory parameters including neutrophil to lymphocyte ratio (NLR) and platelet to lymphocyte ratio (PLR), have attracted extensive attention and have been studied in a wide spectrum of diseases[8–10]. This is because these indexes of systemic inflammation are easy to measure and provide useful information for prognosis [11, 12]. PLR is calculated as the platelet count divided by the lymphocyte count. In the process of tumor angiogenesis, proangiogenic mediators could promote the release of platelets[13]. In addition, antiplatelet agents have been shown to inhibit the growth ability of cancer cells by down-regulating matrix metalloproteinase-9 [14]. Therefore, platelets could reflect the invasive potential of cancer cells to some extent. Lymphocytes, by contrast, are involved in cancer immune surveillance [15]. Lymphocytes also participate in tumor defense by inducing cytotoxic cell death and suppressing the proliferation of tumor cells as well as their maturation [5, 16]. Therefore, the index combining platelets and lymphocytes, PLR, could provide a relatively objective and reliable measurement of the protumor and antitumor effects in patients. Compared with platelets, lymphocytes have a greater involvement in systemic inflammatory responses in patients with cancer and are more easily to be influenced. Therefore, the changes in lymphocyte counts have a more profound influence on PLR. High PLR has been shown to be a potential prognostic indicator in a variety of solid tumors, such as gastric cancer[17], non-small-cell lung cancer[18], breast cancer[19] and hepatocellular carcinoma[20]. In addition, a series of studies were designed to investigate the prognostic value of PLR in colorectal cancer [21–27]; however, the data in these studies presented inconsistent and inconclusive results. Therefore, a comprehensive analysis combining the controversial data is required.

The aim of this study was to provide a systematic and comprehensive evaluation of the prognostic value of PLR in CRC by meta-analysis. We combined results from 13 studies and assessed the prognosis role of PLR for overall survival (OS), disease-free survival (DFS), recurrence-free survival (RFS), cancer-specific survival (CSS), and time to recurrence (TTR) in CRC.

## Materials and Methods

### Literature search

This meta-analysis was performed according to Preferred Reporting Items for Systematic Reviews and Meta-Analyses (PRISMA) guidelines and the PRISMA checklist was shown in S1 PRISMA Checklist. A thorough literature search was performed in the databases of PubMed, Embase, and Web of Science. The latest search was updated on March, 2016. The following terms were used: “PLR or platelet to lymphocyte ratio or platelet-lymphocyte ratio” and “colon cancer or rectal cancer or colorectal cancer or colorectal neoplasms”. References from relevant articles were also examined for possible inclusions.

### Selection criteria

The inclusion criteria were as follows: 1. the diagnosis of CRC was pathologically established; 2. the value of PLR was measured by blood based approaches prior to treatment; 3. information between PLR and clinical outcomes including OS, DFS, RFS, CSS and/or TTR was provided or sufficient data was provided for the estimation of hazard ratio (HR) and 95% confidence interval (CI); 4. a cut-off value to define high PLR was provided; 5. for overlapping studies, the most recent one was selected; 6. the study was published in English. The exclusion criteria were as follows: 1. letters, reviews, meeting abstracts, case reports or nonhuman studies; 2. insufficient data to estimate HRs and 95% CIs; 3. overlapping or duplicate studies.

### Data extraction

Two investigators (XB,G and XS,G) independently extracted the following information from the included studies: first author, publication year, country, study period, sample size, tumor stage, tumor location, PLR cut-off value, treatment methods, and survival analysis. Regarding treatment methods, in the case that all patients received any combination of surgical resection, chemotherapy and radiotherapy treatment, the treatment method was identified as “mixed”. In the event that all of the patients received surgical resection while only a number of them received chemotherapy or radiotherapy, the treatment method was labeled as “surgery”. Any disagreement between the two investigators was settled by discussion.

### Statistical analysis

HRs and 95% CIs were selected to assess the association between PLR and prognosis in CRC. Cochran’s Q test[28] and the Higgins I^2^ statistic[29] were used to estimate heterogeneity. I^2^>50% or P_h_<0.1 indicated significant heterogeneity. Both the random-effects model (DerSimonian Laird method)[30] and fixed-effects model (Mantel Haenszel method)[31] were employed to calculate combined HRs and 95% CIs. In the event that significant heterogeneity was found, the random-effects model was selected to explain the results, while, the fixed-effects model was used. Subgroup analysis stratified by clinical and pathological factors was performed to investigate and interpret heterogeneity between different studies. Publication bias was evaluated by Begg’s funnel plot[32] and Egger’s test[33]. All statistical analyses were performed using STATA V.12.0 (Stata Corp, College Station, TX). P<0.05 was considered as statistically significant.

## Results

### Literature selection and characteristics of included studies

**Fig 1:**
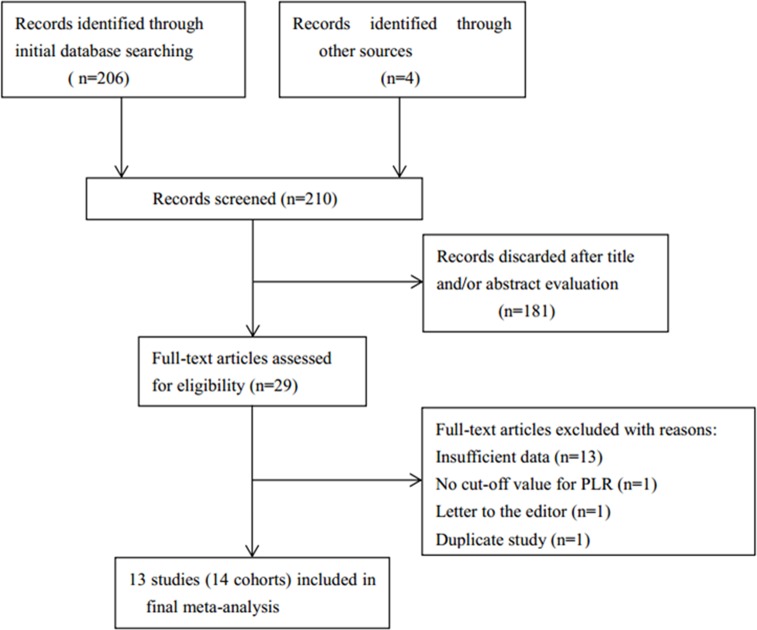
Flow diagram of literature selection.

The initial literature search identified 210 records from the databases of PubMed, Embase, and Web of Science, and reference lists. Subsequent to an evaluation of these records, 181 records were excluded because they were reviews, irrelevant studies, meeting abstracts, duplicate records or animal studies. Therefore, 29 full-text articles were examined for eligibility. Sixteen records were further excluded after analysis of the full-text because they failed to provide key information, did not present a PLR cut-off value, had been published as a letter or were duplicate articles from the same research group. As in Baranyai’s study[23], the investigators recruited 336 patients with CRC and 118 patients with metastatic CRC (mCRC). The CRC group and mCRC group were independent cohorts and were analyzed separately, and thus, we named the CRC cohort ‘Baranyai1’ and the mCRC cohort ‘Baranyai2’. Finally, 13 studies (14 cohorts)[21–27, 34–39] were included in the meta-analysis. The literature selection procedures are shown in Fig 1. All of the included studies had a retrospective study design, and were published between 2012 and 2016. Four studies[26, 37–39] were conducted in China, three [34–36] were performed in Japan, two [21, 24] were carried out in UK, one study was performed in Korea [22], one was from Hungary [23], one was conducted in Austria [25] and one was performed in Canada [27]. The total sample size of the 13 studies (14 cohorts) was 8,601. The main characteristics of included studies were depicted in Table 1.

**Table 1:** Main characteristics of included studies.

Study	Year	Country	Ethnicity	Study period	No. of patients	Gender (M/F)	Tumor stage	Tumor location	Treatment	Cut-off	Follow-up (month)	Survival analysis
Carruthers	2012	UK	Caucasian	2000–2005	115	75/40	Ⅰ-Ⅲ	Rectum	Mixed	160	37.1	OS,DFS,TTR
Son	2013	Korea	Asian	2005–2007	624	368/256	Ⅰ-Ⅲ	Colorectum	Surgery	300	42(1–66)	OS,DFS
Baranyai1	2014	Hungary	Caucasian	2001–2011	336	180/156	Ⅰ-Ⅳ	Colorectum	Surgery	300	36.1	OS,DFS
Baranyai2	2014	Hungary	Caucasian	2001–2011	118	80/38	Ⅳ	Colorectum	Surgery	300	36.1	OS
Neofytou	2014	UK	Caucasian	2005–2012	140	88/52	Ⅳ	Colorectum	Mixed	150	33(1–103)	OS,DFS
Szkandera	2014	Austria	Caucasian	1996–2011	372	217/155	Ⅱ-Ⅲ	Colon	Surgery	176,225^a^	68(1–190)	OS,TTR
Ying	2014	China	Asian	2005–2010	205	144/61	Ⅰ-Ⅲ	Colorectum	Surgrey	176	To Dec,2013	OS,RFS,CSS
Choi	2015	Canada	Caucasian	2004–2012	549	296/253	Ⅰ-Ⅲ	Colorectum	Surgery	295	NA	OS,RFS
Mori	2015	Japan	Asian	2007–2011	157	90/67	Ⅰ-Ⅲ	Colorectum	Surgery	150	20.5(0.2–62.4)	DFS
Ozawa	2015	Japan	Asian	2000–2010	234	142/92	Ⅱ	Colorectum	Surgery	254	64(1–173)	OS,DFS,CSS
Toiyama	2015	Japan	Asian	2001–2012	89	66/23	Ⅰ-Ⅲ	Rectum	Mixed	150	NA	OS,RFS
Li	2016	China	Asian	2007–2014	5,336	3,167/2,169	Ⅰ-Ⅲ	Colorectum	Surgery	219	55.2	OS,DFS
Li	2016	China	Asian	2003–2012	110	58/52	Ⅳ	Colon	Mixed	162	0.9–122	OS
Zou	2016	China	Asian	2006–2012	216	137/79	Ⅰ-Ⅳ	Colorectum	Surgery	246.36	To Jul,2013	OS

OS: overall survival; DFS: disease-free survival; TTR: time to recurrence; CSS: cancer-specific survival; RFS: recurrence-free survival; NA: not available

^a^:176 for TTR, 225 for OS.

### PLR and prognosis for OS

**Fig 2:**
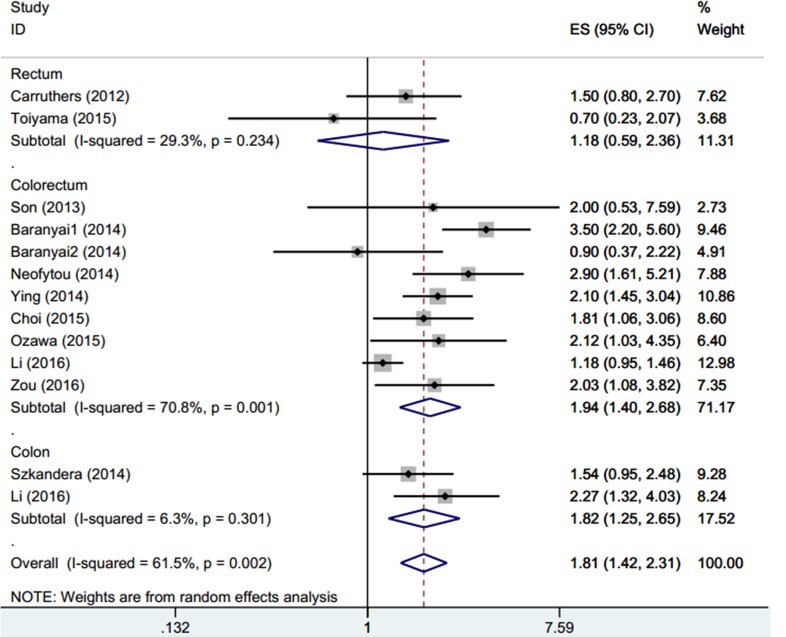
Forest plot of HR for the association between PLR and OS stratified by tumor location in CRC.

A total of 12 studies (13 cohorts)[21–27, 35–39] with 8,444 patients were used to investigate the relationship between PLR and OS in CRC. The pooled HR and 95%CI were HR = 1.81, 95%CI:1.42–2.31, p<0.001 in the random-effects model, with heterogeneity (I^2^ = 65%, P_h_ = 0.002)(Fig 2, Table 2). Subgroup analysis stratified by ethnicity, sample size, tumor location, metastasis status, and treatment demonstrated that PLR had an enhanced prognostic value in Caucasian patients (HR = 1.95, 95%CI: 1.35–2.8, p<0.001; I^2^ = 59%, P_h_ = 0.032), in small sample studies (n<300): HR = 1.94, 95%CI: 1.57–2.4, p<0.001 with moderate heterogeneity (I^2^ = 23.7%, Ph = 0.24) and for those with metastatic disease (HR = 1.98, 95%CI: 1.1–3.55, p = 0.022; I^2^ = 57.1%, P_h_ = 0.097). High PLR also predicted poor OS in CRC (HR = 1.94, 95%CI: 1.4–2.68, p<0.001; I^2^ = 70.8%, P_h_ = 0.001); however, the difference was not significant in rectal cancer (HR = 1.25, 95%CI: 0.75–2.14, p = 0.404; I^2^ = 29.3%, P_h_ = 0.234).

**Table 2:** Summary of the meta-analysis results.

Analysis	No. of studies	Percent (%)	References	No. of patients	Random-effects model		Fixed-effects model		Heterogeneity	
					HR(95%CI)	p	HR(95%CI)	p	I^2^(%)	P_h_
OS										
Overall	13	100	[21–27, 35–39]	8,444	1.81(1.42–2.31)	<0.001	1.65(1.45–1.89)	<0.001	61.5	0.002
Subgroup1: ethnicity										
Caucasian	6	46.15	[21, 23–25, 27]	1,630	1.95(1.35–2.8)	<0.001	2.04(1.62–2.56)	<0.001	59	0.032
Asian	7	53.85	[22, 26, 35–39]	6,814	1.69(1.23–2.32)	0.001	1.48(1.26–1.75)	<0.001	56.9	0.03
Subgroup2: sample size										
>300	5	38.46	[22, 23, 25, 27, 37]	7,217	1.81(1.15–2.86)	0.011	1.48(1.25–1.76)	<0.001	78	0.001
<300	8	61.54	[21, 23, 24, 26, 35, 36, 38, 39]	1,227	1.9(1.48–2.44)	<0.001	1.94(1.57–2.4)	<0.001	23.7	0.24
Subgroup3: tumor location										
Rectum	2	15.38	[21, 36]	204	1.18(0.59–2.36)	0.636	1.25(0.75–2.14)	0.404	29.3	0.234
Colon	2	15.38	[25, 38]	482	1.82(1.25–2.65)	0.002	1.82(1.26–2.61)	0.001	6.3	0.301
Colorectum	9	69.24	[22–24, 26, 27, 35, 37, 39]	7,758	1.94(1.4–2.68)	<0.001	1.66(1.43–1.93)	<0.001	70.8	0.001
Subgroup4: metastasis										
Localized	10	76.92	[21–23, 25–27, 35–37, 39]	8,076	1.76(1.34–2.23)	<0.001	1.59(1.38–1.83)	<0.001	63	0.004
Metastatic	3	23.08	[23, 24, 38]	368	1.98(1.1–3.55)	0.022	2.14(1.48–3.09)	<0.001	57.1	0.097
Subgroup5: treatment										
Mixed	4	30.77	[21, 24, 36, 38]	454	1.85(1.15–2.98)	0.012	1.97(1.43–2.71)	<0.001	50.8	0.107
Surgery	9	69.23	[22, 23, 25–27, 35, 37, 39]	7,990	1.8(1.34–2.41)	<0.001	1.59(1.38–1.84)	<0.001	66.2	0.003
DFS										
Overall	7	100	[21–24, 34, 35, 37]	6,942	1.84(1.22–2.76)	0.003	1.28(1.13–1.46)	<0.001	78.3	<0.001
Subgroup1: ethnicity										
Caucasian	3	42.86	[21, 23, 24]	591	1.93(1.12–3.34)	0.018	1.92(1.45–2.53)	<0.001	72.9	0.025
Asian	4	57.14	[22, 34, 35, 37]	6,351	1.78(0.97–3.26)	0.064	1.15(0.99–1.33)	0.067	69.7	0.019
Subgroup2: sample size										
>300	3	42.86	[22, 23, 37]	6,296	1.84(0.76–4.44)	0.177	1.18(1.02–1.36)	0.028	88.5	<0.001
<300	4	57.14	[21, 24, 34, 35]	646	1.8(1.28–2.54)	0.001	1.77(1.34–2.35)	<0.001	24	0.267
Subgroup3: tumor location										
Rectum	1	14.29	[21]	115	1.2(0.69–2.08)	0.515	1.2(0.69–2.08)	0.515	-	-
Colorectum	6	85.71	[22–24, 34, 35, 37]	6,827	2.01(1.24–3.25)	0.005	1.29(1.13–1.27)	<0.001	81.8	<0.001
Subgroup4: treatment										
Mixed	2	28.57	[21, 24]	255	1.53(1.05–2.23)	0.027	1.55(1.12–2.15)	0.008	22.1	0.257
Surgery	5	71.43	[22, 23, 34, 35, 37]	6,687	2.11(1.11–4.01)	0.023	1.24(1.08–1.43)	0.003	83.9	<0.001
RFS										
Overall	3	100	[26, 27, 36]	843	1.84(1.41–2.41)	<0.001	1.84(1.41–2.41)	<0.001	0	0.686
CSS										
Overall	2	100	[26, 35]	439	1.75(0.59–5.17)	0.309	1.3(0.87–1.96)	0.202	66.2	0.085
TTR										
Overall	2	100	[21, 25]	487	1.21(0.62–2.36)	0.573	1.33(0.91–1.96)	0.145	58.4	0.121

### PLR and prognosis for DFS

**Fig 3:**
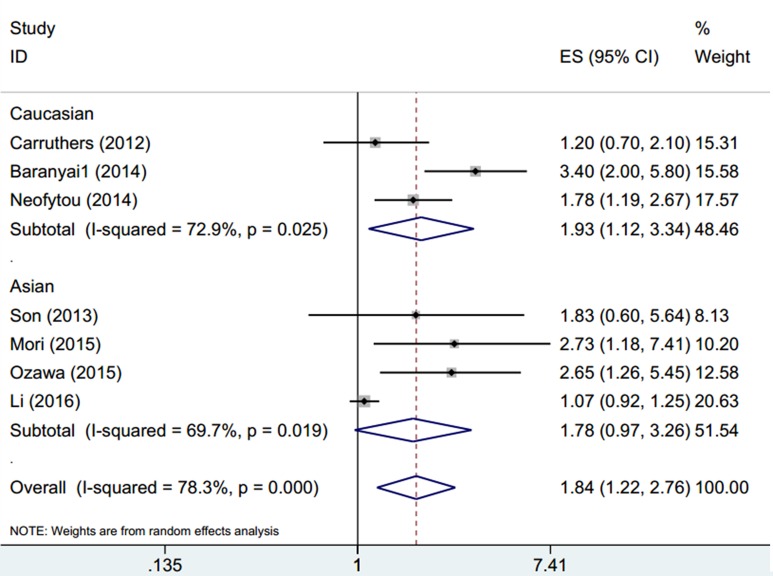
Forest plot of HR for the association between PLR and DFS stratified by ethnicity in CRC.

Seven studies[21–24, 34, 35, 37] involving a total of 6,942 subjects provided the data of PLR for DFS prognosis. The overall HR and 95% CI were HR = 1.84, 95%CI:1.22–2.76, p = 0.003, although with heterogeneity (I^2^ = 78.3%, P_h_<0.001)(Fig 3, Table 2). Stratified analysis suggested that elevated PLR predicted reduced DFS in Caucasian patients (HR = 1.93, 95%CI: 1.12–3.34, p = 0.018; I^2^ = 72.9%, P_h_ = 0.025); however, the result was not statistically significant for Asian patients (HR = 1.78, 95%CI: 0.97–3.26, p = 0.064; I^2^ = 69.7%, P_h_ = 0.019) (Table 2). High PLR was also associated with poorer DFS in patients treated with surgery (HR = 2.11, 95%CI: 1.11–4.01, p = 0.023; I^2^ = 83.9%, P_h_<0.001).

### PLR and prognosis for RFS, CSS, and TTR

Three studies [26, 27, 36] involving a total of 843 patients included data for PLR in RFS. The combined HR and 95%CI were HR = 1.84, 95%CI:1.41–2.41, p<0.001, with fine homogeneity (I^2^ = 0,P_h_ = 0.686). Pooled data from two studies [26, 35] showed that PLR had no statistically significant association with poor CSS in the random-effects model or in the fixed effects model (Table 2). High PLR was also not able to predict poor TTR statistically, according to the pooled HRs and 95% CIs from two articles [21, 25].

### Publication bias

**Fig 4:**
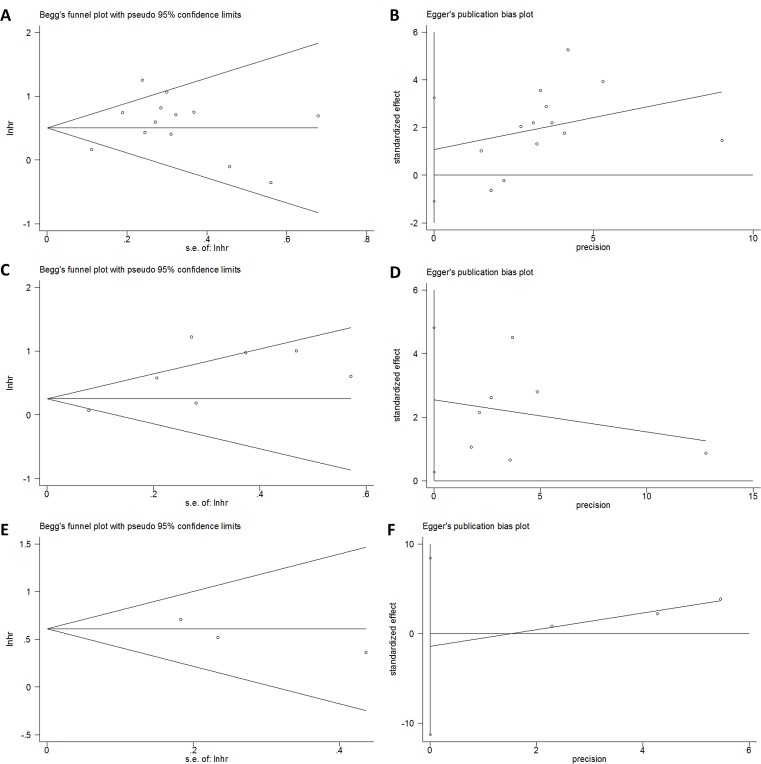
Publication bias assessed by Begg’s test and Egger’s test. (A) Begg’s test for OS; (B) Egger’s test for OS; (C) Begg’s test for DFS; (D) Egger’s test for DFS; (E) Begg’s test for RFS; (F) Egger’s test for RFS.

Begg’s test and Egger’s test were employed to examine the publication bias in the meta-analysis. As shown in Table 3 and Fig 4, the results indicated that there was no significant publication bias present for OS, DFS, RFS, CSS and TTR analyses.

**Table 3:** Publication bias examined by Begg’s test and Egger’s test in meta-analysis.

Variables	No. of studies	Begg’s p	Egger’s p
OS	13	0.36	0.3
DFS	7	0.881	0.064
RFS	3	0.291	0.319
CSS	2	1	-
TTR	2	1	-

## Discussion

In recent years, a series of studies have investigated prognostic role of PLR for CRC, selecting different events, including OS, DFS, RFS, CSS, and TTR as the end-point events. However, these studies have reported conflicting results. While a number of studies[24, 26, 38] suggested PLR to be an effective prognostic biomarker for CRC, other studies[21, 22, 25, 36] reported negative results with respect to the prognostication for PLR. In the present study, by using the meta-analysis analytic approach, we demonstrated that PLR predicted poor OS in CRC, especially in Caucasian patients, for metastatic disease and for CRC; however, it was not able to predict poor OS for rectal cancer. Moreover, a high PLR was correlated with shorter DFS in the overall analysis and had a more significant prognostic value in patients who had received surgery. Furthermore, PLR was also associated with poor RFS, without heterogeneity. However, there was no association between PLR and CSS and TTR in CRC. To the best of our knowledge, this is the first meta-analysis comprehensively exploring the prognostic value of PLR in CRC.

Growing evidence has shown that there is an association between inflammation and tumorigenesis [5, 16]. Recently, tumor-promoting inflammation was established as an emerging hallmark of cancer [7]. Persistence of the inflammatory responses in the tumor microenvironment results in the proliferation of tumor cells, in addition to their metastasis and angiogenesis. Markers of systemic inflammation such as NLR, PLR and C-reactive protein can provide implications for prognosis in CRC [40, 41]. By contrast, prior studies have demonstrated that platelets are involved in the process of tumor angiogenesis [42]. Thrombocytosis is a frequent phenomenon in malignant tumors. A recent study showed that platelet-derived signals were required to guide tumor cells to construct “early metastatic niches”[43]. Furthermore, lymphocytes exert an indispensable role in the antitumor activity of the host by inducing tumor cell apoptosis and by inhibiting tumor metastasis [44]. Moreover, they are able to recognize tumor antigens and exert effects in antitumor responses through mediating antibody-dependent cytotoxicity. Previous studies have reported that an elevation in platelet count is correlated with poor prognosis in colorectal cancer [45, 46]. Current evidence also shows that low tumor-infiltrating lymphocytes are significantly associated with lower 5-year OS and DFS rates in CRC[47]. Based on the biological rationality, the combination of platelets and lymphocytes could be more extensively altered in CRC patients than each one of them. Furthermore, when platelet counts increased and/or lymphocyte counts decreased, the ratio altered more significantly. Moreover, PLR, as a value that combines the platelet and lymphocyte counts, is a more stable indicator of the antitumor status of patients with cancer. In addition, PLR is not difficult to test and involves no added costs, making it suitable to apply in routine clinical settings. Therefore, PLR is a useful and valuable prognostic index.

PLR has been widely explored as a prognostic indicator in various types of cancers. Several meta-analyses have shown that PLR is associated with poor prognosis in patients with non-small-cell lung cancer [48], which was in accordance with our study. Previous studies have also investigated PLR for its predictive role in various solid tumors using meta-analysis [11, 49, 50]. These studies showed that PLR predicted poor prognosis in CRC, in addition to a variety of other tumors. However, the patients with CRC included in the aforementioned studies were limited and subgroup analysis for CRC was not conducted [11, 49, 50]. In the present study, we collected data from 13 studies involving a total of 8,601 patients and combined HRs and 95% CIs in both the random-effects model and fixed-effects model. Furthermore, various end-points for cancer patients including OS, DFS, RFS, CSS and TTR were analyzed in our study. Therefore, our meta-analysis involving patients with CRC is more comprehensive. Interestingly, in the present meta-analysis, we found that PLR was a significant prognostic marker for OS in CRC and colon cancer, but not in rectal cancer. This phenomenon may be due to the fact that different genetic features exist during colon and rectal carcinogenesis [51], and TP53 pathway is activated more frequently in rectal cancer than colon cancer. The activation of platelet-derived growth factor receptor alpha pathway is accompanied by the suppression of p53[52–54], which implies that the elevation of platelet counts may not be significant in rectal cancer carcinogenesis and could even be a protective factor in rectal cancer. Therefore, the increased PLR value was not found to be associated with OS in patients with rectal cancer, as suggested by our results.

Our study did however involve several limitations. Firstly, significant heterogeneity was observed among the included studies. Although we selected primary studies employing uniform inclusion and exclusion criteria, heterogeneity still existed between them. The heterogeneity was possibly due to the various patient ethnicities, different tumor stages, and various treatment methods used in the primary studies. Secondly, publication bias is inevitable in studies; articles with positive results are likely to be published, articles with negative results may not be published. Thus, the combined HR may have been overestimated. Thirdly, the primary studies that reported RFS, CSS, and TTR analysis were limited, so the results concerning RFS, CSS, and TTR should be treated with caution. Therefore, further well-designed and large-scale cohort studies are warranted to confirm the prognostic role of PLR in CRC.

In conclusion, our meta-analysis showed that high PLR was an effective and significant biomarker for poor OS, DFS, and RFS in patients with CRC, however; it did not demonstrate an association with CSS or TTR. Considering the limitations in our study, well-designed, large cohort studies are required to verify our results.

## Supporting Information

S1 PRISMA Checklist (DOC)

S1 File Certificate of English editing from Editage. (PDF)

S2 File STATA program codes. (DOCX)
