# Supplementary material for: Elevated Platelet to Lymphocyte Ratio Is Associated with Poor Survival Outcomes in Patients with Colorectal Cancer
Source: PLoS One. 2016 Sep 22;11(9):e0163523. doi: 10.1371/journal.pone.0163523 (PMC5033452; doi:10.1371/journal.pone.0163523)
Supplement: S1 File — (PDF) [file pone.0163523.s002.pdf]

CERTIFICATE OF ENGLISH EDITING

This document certifies that the paper listed below has been edited to ensure that the language is clear and free of errors. The edit was performed by professional editors at Editage, a division of Cactus Communications.The intent of the author's message was not altered in any way during the editing process. The quality of the edit has been guaranteed, with the assumption that our suggested changes have been accepted and have not been further altered without the knowledge of our editors.

Title Of The Paper

Elevated Platelet to Lymphocyte Ratio is Associated with Poor Survival Outcomes in Patients with Colorectal Cancer

Authors

Xiaobin Gu, Xian-Shu Gao, Shangbin Qin, Xiaoying Li, Xin Qi, Mingwei Ma, Hao Yu, Shaoqian Sun, Dong Zhou, Wen Wang, Wei Xiong

Job Code

NSAOZ_1

editage by CACTUS

Signature

Vikas Narang,
Vice President, Author Services, Editage

Date of Issue
July 22, 2016

Editage, a brand of Cactus Communications, offers professional English language editing and publication support services to authors engaged in over 500 areas of research. Through its community of experienced editors, which includes doctors, engineers, published scientists, and researchers with peer review experience, Editage has successfully helped authors get published in internationally reputed journals. Authors who work with Editage are guaranteed excellent language quality and timely delivery.

CACTUS®

Contact Editage

Worldwide	Japan	Korea	China	Brazil	Taiwan
request@editage.com	submissions@editage.com	submit-korea@editage.com	fabiao@editage.cn	inquiry.brazil@editage.com	submitjobs@editage.com
+1 877-334-8243	+81 03-6868-3348	1544-9241	400-005-6055	0800-892-20-97	02 2657 0306
www.editage.com	www.editage.jp	www.editage.co.kr	www.editage.cn	www.editage.com.br	www.editage.com.tw
