# Supplementary material for: Elevated Platelet to Lymphocyte Ratio Is Associated with Poor Survival Outcomes in Patients with Colorectal Cancer
Source: PLoS One. 2016 Sep 22;11(9):e0163523. doi: 10.1371/journal.pone.0163523 (PMC5033452; doi:10.1371/journal.pone.0163523)
Supplement: S2 File — (DOCX) [file pone.0163523.s003.docx]

S2 File. STATA program codes.

**All statistical analyses were performed using STATA V.12.0 (Stata Corp, College Station, TX). P<0.05 was considered as statistically significant.**

1 **For pooled HR and 95%CI calculation, subgroup analysis stratified by ethnicity, random-effects model:**

metan lnhr lnll lnul, label(namevar=study, yearvar=year) by(ethnicity) random eform

2 **Heterogeneity among studies calculation:**

Heterogeneity calculated by formula

Q = SIGMA_i{ (1/variance_i)*(effect_i - effect_pooled)^2 }

where variance_i = ((upper limit - lower limit)/(2*z))^2

Our original input were hazard ratio (hr), lower limit(ll) and upper limit(ul), which were obtained from the Cox regression in the primary studies. For example, in Neofytou’s study (Ref. 24), in Table 3, the multivariate analysis for PLR on OS, the results were: HR= 2.17, 95%CI= 1.09–4.32, p=0.027. So we extracted hr=2,17, ll=1.09, ul=4.32 for this study. Then we calculated lnhr, lnll and lnul according to the original data using STATA V.12.0 and afterwards the subsequent calculations.

**In this meta-analysis including 14 studies, Begg and Mazumdar bias test should be treated with caution. Because “The test is fairly powerful for large meta-analyses with 75 component studies, but has only moderate power for meta-analyses with 25 component studies. Even 25 studies is a pretty large meta-analysis.”** (Begg, C. B. and Mazumdar, M., 1994, PMID: 7786990)

**3 Tests for Publication Bias**

metabias lnhr selnhr, graph(begg)

metabias lnhr selnhr, graph(egger)
